# A Comparative Study on the Cultural Dimensions and Health Perception of the COVID-19 Pandemic between China and the United States

**DOI:** 10.3390/healthcare10061081

**Published:** 2022-06-10

**Authors:** Yahan Zhang, Shanshan Liu, JongWoo Jun

**Affiliations:** 1 Department of Communication, Dankook University, Yongin-si 16890, Korea; yahanz659@gmail.com; 2 School of Journalism and Communication, Pingdingshan University, Pingdingshan 467099, China; 3 School of Communication, Nanyang Institute of Technology, Nanyang 473004, China; shanshan892@hotmail.com

**Keywords:** cultural differences, health perception, risk perception, seriousness, prevention behavioral intention

## Abstract

When a public crisis such as COVID-19 occurs, factors that affect health-related behaviors, such as compliance with safety precautions, health professionals, and directives from government agencies will become more obvious. This research explores the differences between the people of the United States and China regarding preventive behavioral intentions, perceptions of personal and social risks, seriousness, and other cultural characteristics in the context of the COVID-19 health crisis. The purpose is to provide insights that can be used when global public health events occur in the future. A total of 536 people who lived in the US and China from 12 July to 7 September 2020 were recruited in the survey. Through a web-based survey, differences in the attitudes and perceptions of COVID-19 between the two countries were identified. Overall, the people of China scored higher than Americans on several measures regarding personal risk perception, social risk perception, and seriousness. Chinese citizens also had higher preventive behavioral intentions than their US counterparts. In addition, the relationships between cultural dimensions and health-related variables were also different.

## 1. Introduction

Since the global outbreak of COVID-19 began at the end of 2019 to the beginning of 2020, the pandemic has had a tremendous impact on the global economy. While bringing extreme changes to people’s lives, it also has altered the lifestyle and work of many ordinary people [1]. The current epidemic situation in China is mostly under control, and there are basically no new confirmed cases. After the popularization of the COVID-19 vaccine, people will be able to return to a normal lifestyle; however, small-scale epidemics in provinces and cities will still occur. Wu Liangyou, deputy director of the National Health Commission’s Bureau of Disease Control and Prevention, stated that, as of 7 June 2022, a total of 3,385.853 million doses of the COVID-19 vaccine have been reported nationwide. The main brands of COVID-19 vaccine in China are Sinopharm (Vero Cell, inactivated), CoranaVac (SARS-CoV-2 vaccine (Vero Cell), inactivated), CanSino COVID-19 vaccine, Zifivax COVID-19 vaccine, etc. With the boost in vaccination rates in the United States, the degree of epidemic prevention and control is being gradually relaxed. Although the number of daily cases fluctuates, numerous schools have resumed offline teaching, and the people are restoring their pre-pandemic lifestyles. As of 6 May 2022, 77.7% of the population in the United States had been vaccinated at least once, and 66.28% had completed the vaccination process [2]. The main brands of COVID-19 vaccine in United States are the Moderna COVID-19 vaccine, Pfizer/BioNTech COVID-19 vaccine, AstraZeneca COVID-19 vaccine, etc.

The findings of Zhou, Ghose, and Wang confirm that the Chinese survey participants have a high health awareness of COVID-19 and are optimistic about successfully fighting the epidemic [3]. From a cultural perspective, Luo et al. analyzed the perceptions of American and Chinese social media users on vaccination policies, priority groups, challenges arising from COVID-19 variants, and themes of the global pandemic situation through a semantic network. They found that the United States of America Twitter users tend to disclose personal vaccination experiences and express antivaccination attitudes. Contrastingly, Weibo users in China comply with the authorities. Additionally, Chinese social media users expressed more positive feelings about the COVID-19 vaccine, compared with their US counterparts [4]. In Bruine de Bruin’s study, age differences in risk perceptions and mental health in the early stages of the COVID-19 outbreak in the United States were examined. He concluded that relatively older US adults appeared to have a more optimistic outlook and better mental health in the early stages of the pandemic [5].

Despite extensive literature delineating different aspects of health cognition in the context of the COVID-19 pandemic, few studies delve into the comparison between China and the United States. In view of this research gap, in this study, we analyze the health perception of COVID-19 in China and the United States from a cultural perspective and explore the differences in American and Chinese public perceptions of individual and societal risk perception, seriousness, and prevention behavioral intentions, in order to guide the future epidemic control of the two countries and provide a reference for future global public health tragedies.

## 2. Materials and Methods

### 2.1. Setting and Participants

The survey was conducted from 12 July to 7 September 2020 in two densely populated cities in China and the United States. The survey respondents are urban residents aged 20–70 who have a basic understanding of the COVID-19 and have a bachelor’s degree or above, covering various occupational fields. The subjects were selected by random sampling and participated voluntarily through online questions using Questionnaire Star, an international research company with a broad client base and extensive research experience. The target comprises 5,331,323 people aged 20–70. Therefore, a sample of at least 384 individuals was estimated to evaluate the selected variables, assuming a 5% margin of error and a response proportion of 50%, with a 95% confidence level [6,7].

### 2.2. Ethical Statement

To comply with academic ethics, the consent and approval of the interviewees were obtained in advance of field research and interviews. When referring to individuals, plantations, and companies, we used pseudonyms to protect the anonymity of the respondents.

### 2.3. Theoretical Background and Measurement

#### 2.3.1. Cultural Differences

Hofstede’s IBM study is one of the most cited studies explaining cultural differences among countries. This early research involved a survey of IBM employees in the early 1970s [8]. Hofstede was working in IBM’s HR department at the time. He found that, depending on the cultural environment, the degree to which subordinates communicated without notifying their supervisor was different. Based on this observation, he conducted ethnic training for IBM employees around the world. This was one of the first comparative studies of cultural differences [9]. It revealed five cultural dimensions—namely, power distance (PD), uncertainty avoidance (UA), individualism (IDV), long-term orientation (LTO), and masculinity (MAS).

Power refers to the probability that an individual can realize his will even when he encounters severe resistance from others or organizations [10]. Uncertainty avoidance refers to the degree of threat people feel when faced with uncertainty and unknown situations. In order to avoid uncertainties, people will adopt behavioral activities including technology, religion, and the law to cope [9]. The framework regarding organizational behavior proposed by Hofstede in 1980 is very representative. He initially defined individualism/collectivism as the degree to which people care about group members and group goals or their own, and personal goals [9]. Long-term orientation/short-term orientation is a dimension in the cultural framework proposed by Hofstede, which originated from the Confucian work dynamism in research from Bond and Feather [11]. The concept of masculinity and femininity was first proposed by Terman and Miles in 1936 [10]. It does not simply refer to the physical differences between men and women, but the different characteristics of men and women in gender roles. Hofstede’s theory attributed professional competition, self-confidence, courage, and the desire for success to masculinity, while gentleness, tolerance, and caring for the family endow femininity [8]. Table 1 for a review of studies on the cultural dimension in China and the United States.

Five cultural dimensions of Hofstede were measured with the scales from Wang and Zhang [12]. The statements of PD were “*People in higher positions should make most decisions without consulting people in lower positions”*; “*People in higher positions should not ask the opinions of people in lower positions too frequently*”; “*People in higher positions should avoid social interaction with people in lower positions*”; “*People in lower positions should not disagree with decisions by people in higher positions*”; and “*People in higher positions should not delegate important tasks to people in lower positions*”.

The statements to measure UA were “*It is important to have instructions spelled out in detail so that I always know what I’m expected to do*”; “*It is important to closely follow instructions and procedures*”; “*Rules and regulations are important because they inform me of what is expected of me*”; “*Standardized work procedures are helpful*”; and “*Instructions for operations are important*”.

IDV levels were measured by the following statements: “*Individuals should sacrifice self-interest for the group*”; “*Individuals should stick with the group even through difficulties*”; “*Group welfare is more important than individual rewards*”; “*Group success is more important than individual success*”; “*Individuals should only pursue their goals after considering the welfare of the group*”; and “*Group loyalty should be encouraged even if individual goals suffer*”.

To measure LTO, the following statements were used: “*Careful management of money*”; “*Going on resolutely in spite of opposition*”; “*Personal steadiness and stability*”; “*Long-term planning*”; “*Giving up today’s fun for success in the future*”; and “*Working hard for success in the future*”.

The statements to measure MAS were “*It is more important for men to have a professional career than it is for women*”; “*Men usually solve problems with logical analysis; women usually solve problems with intuition*”; “*Solving difficult problems usually requires an active, forcible approach, which is typical of men*”; and “*There are some jobs that a man can always do better than a woman*”.

#### 2.3.2. Health Perception

One of the main factors influencing people’s decision to make the corresponding health protection behavior is the individual’s perception of health [21]. Health perception is linked to the evaluation status of past diseases or medical experiences. Therefore, it is more subjective than objectively measured clinical examination results. It is regarded as one of the universal health indicators for evaluating health levels [22]. Ware believes that the measurement of health perception includes self-assessments of health, feelings of well-being, energy levels, and vitality, which is an evaluation standard when medical methods cannot accurately measure health status [23]. Liu believes that the level of health perception is determined by its own and social factors. Its own factors include the type of household registration, education level, social status, occupation, etc. In contrast, social factors include developing and popularizing health education and health promotion, basic public health services, and media publicity and guidance [24]. Through the combination of existing research, in this study, we chose personal risk perception/social risk perception (PRP/SRP), seriousness (SER), and prevention behavioral intentions (PBI) as variables to gauge health perception.

Risk perception is formed by judgments on various types of information that estimate the extent to which risks will affect them [25]. In this study, we sought to find differences in risk perception between the United States and China. Given that personal risk perception and social risk perception are different, it is more desirable to explore both risk perceptions independently.

The public tends to evaluate risk based on the seriousness of the results after the risk occurs, and the seriousness of public perception depends on fear [26]. Therefore, we focused on the perception of seriousness and examines the differences between the United States and China.

Behavioral intention explains changes in human social actions and is considered to be the most timely and important determinant of behavior [27]. To form a PBI, the most important aspect is to perceive that the danger is related to oneself and that the possibility of this danger actually happening to oneself is real. Many studies have confirmed that this risk perception is the most important factor in PBI [28]. Table 2 for A review of studies on health perception.

Risk perception was measured by the scales of Morton and Duck [35]. PRP for this study was measured with the following statements: “*COVID-19 is important problem for me*”; “*I am concerned about the damage I will receive from COVID-19*”; “*I will be damaged by COVID-19*”; “*I personally feel the risk of COVID-19”*. SRP was measured with the following statements: “*COVID-19 is important problem for the community*”; “*The public worry about the damage they will receive from COVID-19*”; “*The public will be harmed by COVID-19*”; and “*The public has a high risk of feeling about COVID-19”*.

SER was measured by Rimal et al. [36]. In this study, we used the following statements: “*COVID-19 is a serious risk that someone could die*”; “*If someone is exposed to COVID-19, it is very likely to cause serious damage*”; “*COVID-19 is one of the serious diseases*”; and “*COVID-19 is more deadly than any other disease*”.

PBI was measured by the following statements: “*I will actively put social distances if necessary*”; “*I will actively wear a mask if necessary*”; and “*I will actively participate in hand washing if necessary*”.

Both the Chinese and English questionnaires were proofread by professionals who are native speakers of these two languages, which ensured that the contents of the two questionnaires are completely consistent, so they have good intelligibility for both Chinese and Americans. Subjects were asked to answer these questions on a 7-point scale (1 = Not at all; 7 = A lot).

### 2.4. Research Questions

Based on the discussed cultural dimensions and health perception, we summarized individual definitions of each cultural dimension and traditional differences between the United States and China. However, cultural orientation and health perception could be changed, as the social environments of the two nations are different. In this regard, we proposed 5 research questions exploring the differences between the United States and China (Table 3). 

### 2.5. Statistical Analysis

In our methodology, we combined a review of the literature with quantitative analysis, to measure the health perception of the COVID-19 pandemic in China and the United States. One-way ANOVA and regression analysis were used for data analysis, and the statistical package used for the analysis was SPSS. The ANOVA is a parametric statistical technique used for the comparison of group means. In this study, it was used to compare the differences in culture and health perception between American and Chinese subjects. Regression analysis refers to a statistical analysis method to determine the interdependent quantitative relationship between two or more variables. It was used in this study to assess the impact of culture-related variables on health perceptions among American and Chinese subjects.

## 3. Results

From a demographic perspective, of the 536 total participants, 313 (58.4%) were in the United States, and 223 (41.6%) were in China. There were 267 (49.8%) females and 269 (50.2%) males, with an age range spanning from 20 to 70, and a mean of 40.0 (SD = 11.2) (Table 4).

Reliability reflects the stability and consistency of measurement. Cronbach’s α coefficient was used to test the consistency of the subjects’ answers to the questions on the previously mentioned scale. The test results showed that Cronbach’s α coefficient of each variable ranged from 0.712 to 0.919, which is higher than the acceptable standard of 0.6. Therefore, the reliability of the scale is relatively high. Validity reflects the authenticity of measurement, and the validity of a scale is measured by content validity and convergent validity. The results showed that the index of the KMO coefficient was 0.876, the result of Bartlett’s test of sphericity was 5754.630 (*p* = 0.000), and the factor loading value of each dimension was greater than 0.7.

### 3.1. ANOVA

For research question 1, regarding the power distance index, there was a statistically significant difference between American and Chinese subjects, as determined by a one-way ANOVA (Table 5). It was found that Chinese subjects had higher scores (M = 2.9265, SD = 1.61434) than American subjects (M = 1.8497, SD = 0.77461). Regarding uncertainty avoidance, there was a statistically significant difference between American and Chinese subjects, as determined by a one-way ANOVA. It was found that Chinese subjects had higher scores (M = 5.2179, SD = 1.25993) than American subjects (M = 2.8508, SD = 1.22168). Regarding individualism/collectivism, there was a statistically significant difference between American and Chinese subjects, as determined by a one-way ANOVA. It was found that Chinese subjects had higher scores (M = 3.7549, SD = 1.4581) than American subjects (M = 3.1405, SD = 1.17798). Regarding long-term orientation, there was a statistically significant difference between American and Chinese subjects, as determined by a one-way ANOVA. It was found that Chinese subjects had higher scores (M = 5.4178, SD = 1.00510) than American subjects (M = 2.7018, SD = 1.09859). Regarding masculinity, there was a statistically significant difference between American and Chinese subjects, as determined by a one-way ANOVA. It was found that Chinese subjects had higher scores (M = 3.6883, SD = 1.57949) than American subjects (M = 3.2903, SD = 1.137465).

In addition, a separate one-way ANOVA was conducted between American and Chinese subjects on the four variables of personal risk perception, social risk perception, seriousness, and preventive behavior intention (Table 6). For research question 2-1, regarding personal risk perception, there was a statistically significant difference between American and Chinese subjects, as determined by a one-way ANOVA. It was found that Chinese subjects had higher scores (M = 5.5662, SD = 1.24525) than American subjects (M = 2.8379, SD = 1.10794). Regarding social risk perception, there was a statistically significant difference between American and Chinese subjects, as determined by a one-way ANOVA. It was found that Chinese subjects had higher scores (M = 6.0964, SD = 0.95224) than American subjects (M = 2.4870, SD = 1.03800).

For research question 3, regarding seriousness, there was a statistically significant difference between American and Chinese subjects, as determined by a one-way ANOVA. It was found that Chinese subjects had higher scores (M = 5.8296, SD = 1.16736) than American subjects (M = 2.7225, SD = 1.08863).

For research question 4, regarding preventive behavioral intention, there was a statistically significant difference between American and Chinese subjects, as determined by a one-way ANOVA. It was found that Chinese subjects had higher scores (M = 6.3934, SD = 0.84518) than American subjects (M = 2.3344, SD = 1.15609).

### 3.2. Regression

Regression analysis was employed to identify links between cultural dimension and risk perception, seriousness, and behavioral intention (Table 7). The *p*-values of independent variables of LTO (t = 24.443, *p* = 0.000) on the dependent variable of PRP was 0.000, meeting the standard value *p*-value of less than 0.05. Therefore, LTO positively impacted PRP. Moreover, LTO (t = 16.213, *p* = 0.000), MAS (t = −3.769, *p* = 0.000), PD (t = 2.841, *p* = 0.005), and UA (t = 2.782, *p* = 0.006) significantly influenced the dependent variable SRP. LTO, PD and UA positively impacted SRP, whereas MAS negatively impacted SRP. When SER was the dependent variable, LTO (t = 11.473, *p* = 0.000), PD (t = 2.947, *p* = 0.003), and UA (t = 2.215, *p* = 0.027) as independent variables affected SER values. LTO, PD, and UA had positive impacts.

When PBI was the dependent variable, LTO (t = 17.271, *p* = 0.000), PD (t = 4.317, *p* = 0.000), and UA (t = 2.887, *p* = 0.004) had positive effects on it, and MAS (t = −5.128, *p* = 0.000) negatively influenced PBI.

The analysis results for the United States demonstrate that LTO (t = 7.956, *p* = 0.000) and PD (t = 3.786, *p* = 0.000) positively impacted PRP, whereas LTO (t = 8.657, *p* = 0.000) and MAS (t = −2.700, *p* = 0.007) influenced SRP. Among these variables, only MAS had a negative influence relationship, and LTO and COL had positive influences. Moreover, LTO (t = 4.073, *p* = 0.000) positively impacted SER. Finally, LTO (t = 4.073, *p* = 0.000), PD (t = −2.964, *p* = 0.003), and UA (t = 2.498, *p* = 0.013) affected PBI, and PD had a negative impact. Additionally, both LTO and UA had positive impacts (Table 8).

The analysis for China revealed that LTO (t = 4.023, *p* = 0.000) positively impacted PRP. LTO (t = 7.298, *p* = 0.000) and PD (t = −3.201, *p* = 0.002) had effects on SRP, of which LTO had a positive impact, and PD had a negative impact. LTO (t = 4.820, *p* = 0.000) had a positive effect on SER. LTO (t = 7.025, *p* = 0.000) and MAS (t = −2.092, *p* = 0.038) influenced PBI—specifically, LTO had a positive impact, and MAS had a negative impact (Table 9).

## 4. Discussion

Through a survey of 563 users, cultural differences were found between American and Chinese subjects (R.Q. 1). Overall, the results showed that Chinese subjects had a higher sense of personal risk, social risk, and seriousness of COVID-19 than American subjects (R.Q. 2 and R.Q. 3). In addition, the results found that Chinese subjects had higher preventive behavioral intention than American subjects (R.Q. 4). It was also found that the relationships between cultural dimensions and health perception differed between participants from China and the United States (R.Q. 5).

Studies on the cultural dimension have proved that the Chinese have a higher power distance and are more long-term-oriented than Americans. This finding is consistent with the cultural tendency expressed in the existing Hofstede’s cultural dimension index [8], also aligning with the findings of Wang [14] and Wang [9]. However, in this study, Chinese subjects appeared to be more masculine than Americans. American and Chinese subjects showed similar levels of masculinity in Hofstede’s cultural index; however, Chinese subjects showed a more masculine tendency than American subjects in this study; this finding is similar to Yin’s results on Chinese state-owned enterprises [12]. Moreover, Chinese subjects scored higher than the American subjects in individualism and uncertainty avoidance. The findings of Yin also confirmed that small private companies in China have lower risk resistance and stronger uncertainty avoidance, which are consistent with the findings of this study [12]. However, contrary to the findings of this study, in the face of the COVID-19 pandemic, Chinese subjects’ individualism exceeds that of the American subjects. Moreover, from a socio-cultural perspective, Americans possess high individualistic culture, while the Chinese live in a country with typical collectivist characteristics [13].

Delving into the influence of the cultural dimension on health-related perceptions, long-term orientation appeared to have the most significant influence. Dependent variables and cultural factors all seemed to have a greater influence on social risk perception than individual risk perception. All cultural dimensions influenced social risk perception and the preventive behavior intention. All cultural dimensions except MAS influenced the perception of seriousness. The analysis of the direction of the variables revealed that MAS had a negative effect, and individuals with individualistic tendencies were active in social risk perception, seriousness perception, and preventive action intention.

Moreover, differences between American and Chinese subjects were noted. In both the United States and China, long-term orientation affected all variables despite differences in other cultural dimensions. Only power distance and masculinity were additionally significant in China. Power distance negatively impacted social risk perception; consequentially, people with low power distance had higher social risk perception. Masculinity negatively impacted preventive behavioral intentions. As a result, feminine-oriented people were found to actively participate in preventive behavioral intentions. On the other hand, in the case of the United States, more diverse cultural dimensions exerted an influence. Specifically, collectivism affected social risk perception and seriousness perception, while participants with a feminine tendency had a higher perception of social risk. Those with high uncertainty avoidance tended to have higher preventive behavioral intentions.

The research on health perception in this study confirmed that there are fundamental differences in terms of attitude and perceptions regarding COVID-19 between American and Chinese subjects. Chinese subjects had higher personal risk perception, social risk perception, and concern about COVID-19 than American subjects. Chinese subjects also had higher preventive behavioral intentions than American subjects. This result slightly differs from the results of Han et al., as they did not find significant differences in risk perception between China and the United States [27].

### Limitations of the Study

The main limitations of this study are as follows: Firstly, the sampling used in this study can be criticized. The United States and China are both large nations; in particular, China’s population is among the largest in the world. Therefore, considering that the overall sample size surveyed is small, it may not be a representative sample of the population in each country. Secondly, in terms of demographic characteristics, factors such as age, occupation, education level, and income of the respondents should be considered against factors that may affect public judgment this may be included in future research. In future research, a larger sample would allow the research and conclusion to be more generalizable. In addition, future research dealing with other countries representing Western or Eastern cultures will be necessary.

## 5. Conclusions

Based on the above results, we attempted to analyze the reasons behind the differences found in this study from the following aspects:

Firstly, the development and changes in cultural dimensions through the research results are conspicuous. China is often classified as a collectivist country and is recognized as possessing an Asian cultural identity [16]. However, the results of this study proved that modern China is more individualistic than the United States. This change may prove that the characteristics of the country in the cultural dimension will change, and may likely be due to the influence of the objective external environment, such as other natural disasters or epidemics; this also arises due to internal factors such as national economic development and the continuous improvement of people’s cognition level.

Secondly, after sorting the results of previous studies, it was found that few studies compared the differences in seriousness and prevention behavioral intentions between China and the United States. Therefore, it is difficult to find the laws of these two variables between the two countries from the existing studies. From the results of this study, it was revealed that there were obvious differences between American and Chinese subjects across these four variables, and the average values of Chinese subjects markedly exceeded those of the American subjects. In the context of COVID-19, Chinese people are very strong in personal risk perception and social risk perception, not only in seriousness but also in prevention behavioral intentions. The reason for this result may be found in the influence of the cultural dimensions on health perception. Since the Chinese are generally long-term-oriented, Chinese subjects in this study had high levels of power distance index and masculinity tendency. During large-scale public health events, people are more sensitive to risk perception and closely consider the seriousness that such events may entail. Therefore, they are more likely to produce corresponding prevention behavioral intentions. Contrastingly, for American subjects in this study, the influence of cultural dimensions on health perception was more dispersed, thereby weakening the influence of a single dimension on health perception to a certain extent. These findings also reflect the characteristics of American cultural diversity.

## Figures and Tables

**Table 1:** A review of studies on cultural dimensions in China and the United States.

Cultural Dimensions	Author Name and Year	Main Points
Power Distance	Yin Q., 2014	The power distance of Chinese state-owned enterprises is significantly inferior to that of South Korean and American companies [12].
	Tegethoff I., 2022	Chinese series promotes a greater tolerance of hierarchical structures and a partially closer social distance in asymmetrical professional relationships than American and German. These disparities are related to different perceptions of power distance, role relationships, face, and harmony [13].
	Wang Y. H., 2014	Chinese parents have stronger control over their children and they have an uneven distribution of power, compared with their American counterparts [14].
Uncertainty Avoidance	Yin Q., 2014	Most Chinese private companies are small in scale and have the lowest ability to take risks or encounter emergencies, compared with those in the United States and South Korea [12].
Individualism	Jin L. H., 2022	Whether it is from the translation of the brand name, the packaging of the product, the choice of words in advertising and promotional slogans, or the difference in situation creation, the marketing strategies of Mondelez’s brands in Western countries reflect the characteristics of individualism. By contrast, after the founding of the People’s Republic, the characteristics of “collectivism” have become more prominent in China [15].
	Zhang X., 2021	By comparing the differences between China and the United States in the four aspects of socialization, work, social relationships, and motivation, it was found that Chinese values are more individualistic than American values in terms of socialization and social relationships [16].
	Yin Q., 2014	Chinese state-owned enterprises have obvious collectivist tendencies; however, private enterprises show individualistic tendencies similar to those of South Korean and American companies [12].
Long-term Orientation	Wu S. and Joardar, A., 2012	Entrepreneurs in China may be more focused on their actions and more persistent in what they accomplish, while American entrepreneurs are more vulnerable to changes in external conditions. Therefore, the differences between Chinese and American entrepreneurs may widen as the entrepreneurial process progresses [17].
	Yin Q., 2014	Chinese state-owned enterprises have a clear long-term tendency, while the long-term tendency of American companies is low [12].
	Wang W., Yang J. H., and Wang K. L., 2018	The long- and short-term orientation dimensions can better fit the continuous bilateral relationship between China and the United States [18].
Masculinity	Zheng L., Lippa R. A., and Zheng Y., 2011	In both Chinese and Western cultures, gender-related interests show significant gender differences. Men prefer masculine hobbies and women prefer feminine hobbies [19].
	Yin Q., 2014	Chinese state-owned enterprises have a higher masculinity tendency, while Chinese private enterprises, South Korean enterprises, and American enterprises all have a higher femininity tendency [12].
	Han G., Shen G. L., and Chu K. J., 2013	Overall risk perception of influenza vaccine efficacy did not differ significantly between China and the United States [20].

**Table 2:** A review of studies on health perception.

Health Perception	Author Name and Year	Main Points
Personal Risk Perception/Social Risk Perception	Hsieh Y. C. J., Chen Y. L., and Yin P., 2022	US respondents are more concerned about privacy, legal, and liability risks than their Chinese counterparts, while Chinese respondents are more concerned about social risks, device risks, and performance/satisfaction risks than their US counterparts [29].
	Han G., Shen G. L., and Chu K. J., 2013	Overall risk perception of influenza vaccine efficacy did not differ significantly between China and the United States [20].
Seriousness	Altheide D. L., 2020	The official position of the United States was to downplay the seriousness of the deadly virus [30].
	Christensen T. J., 2020	The Chinese government takes the contagiousness and seriousness of the COVID-19 more seriously than other countries [31].
	Song H. R., Kim C.W., Kim W. J., 2014	Risk perception formed through various experiences can impact predicting or assessing the seriousness of risk [32].
Prevention Behavioral Intentions	Bae S. Y. and Chang P. J., 2021	Despite the significant influence of both cognitive and affective risk perceptions on behavioral intention, affective risk perception exerts a negative influence on behavioral intention [33].
	Azadi Y., Yazdanpanah M., and Mahmoudi H., 2019	Beliefs had no significant effects on risk perception and adaptation behavior, and trust and risk perception had direct positive effects on adaptation behavior [34].

**Table 3:** Summary of research questions.

Research Question 1 (R.Q. 1)	What are the cultural differences between the American and Chinese subjects?
Research Question 2 (R.Q. 2)	2-1: What is the difference in PRP between the American and Chinese subjects?
	2-2: What is the difference in SRP between the American and Chinese subjects?
Research Question 3 (R.Q. 3)	What is the difference in SER between the American and Chinese subjects?
Research Question 4 (R.Q. 4)	What is the difference in PBI between the American and Chinese subjects?
Research Question 5 (R.Q. 5)	What are the relationships between cultural dimensions and health perceptions?

**Table 4:** Sample characteristics.

Variables	*n* (%)/Mean (SD)
United States	313 (58.4%)
China	223 (41.6%)
Male	269 (50.2%)
Female	267 (49.8%)
Age	40.0 (11.2)

**Table 5:** Cultural differences between the US and China.

DV	IV	M	SD	N	M.S.	F	Sig.
PD	The US	1.8497	0.77461	298	147.893	101.428	0.000
	China	2.9265	1.61434	223			
UA	The US	2.8508	1.22168	295	711.585	464.074	0.000
	China	5.2179	1.25993	223			
IDV	The US	3.1405	1.17798	293	47.793	28.004	0.000
	China	3.7549	1.45819	223			
LTO	The US	2.7018	1.09859	299	942.250	839.091	0.000
	China	5.4178	1.00510	223			
MAS	The US	3.2903	1.37465	304	20.381	9.499	0.002
	China	3.6883	1.57949	223			

PD = power distance, UA = uncertainty avoidance, IDV = individualism, LTO = long-term orientation, MAS = masculinity.

**Table 6:** Health perception differences between the US and China.

DV	IV	M	SD	N	M.S.	F	Sig.
PRP	The US	2.8379	1.10794	307	951.413	698.563	0.000
	China	5.5662	1.24525	219			
SRP	The US	2.4870	1.03800	307	1682.858	1673.350	0.000
	China	6.0964	0.95224	223			
SER	The US	2.7225	1.08863	309	1250.442	992.799	0.000
	China	5.8296	1.16736	223			
PBI	The US	2.3344	1.15609	313	2139.836	1983.999	0.000
	China	6.3934	0.84518	222			

PRP = personal risk perception, SRP = social risk perception, SER = seriousness, PBI = prevention behavioral intentions.

**Table 7:** The impact of cultural differences on health perception between the US and China.

DV	IV	Beta	t	Sig.
PRP	LTO	0.748	24.443	0.000 ***
SRP	LTO	0.781	16.213	0.000 ***
	MAS	−0.119	−3.769	0.000 ***
	PD	0.087	2.841	0.005 **
	UA	0.136	2.782	0.006 **
SER	LTO	0.668	11.473	0.000 ***
	PD	0.101	2.947	0.003 **
	UA	0.131	2.215	0.027 *
PBI	LTO	0.791	17.271	0.000 ***
	MAS	−0.153	−5.128	0.000 ***
	PD	0.125	4.317	0.000 ***
	UA	0.134	2.887	0.004 **

*** *p* < 0.001, ** *p* < 0.01, * *p* < 0.05. PD = power distance, UA = uncertainty avoidance, LTO = long-term orientation, MAS = masculinity, PRP = personal risk perception, SRP = social risk perception, SER = seriousness, PBI = prevention behavioral intentions.

**Table 8:** The impact of cultural differences on health perception in the US.

DV	IV	Beta	t	Sig.
PRP	LTO	0.439	7.956	0.000 ***
	PD	0.209	3.786	0.000 ***
SRP	LTO	0.533	8.657	0.000 ***
	MAS	−0.185	−2.700	0.007 **
	LTO	0.269	4.073	0.000 ***
PBI	LTO	0.529	6.944	0.000 ***
	PD	−0.146	−2.964	0.003 **
	UA	0.194	2.498	0.013 *

*** *p* < 0.001, ** *p* < 0.01, * *p* < 0.05. PD = power distance, UA = uncertainty avoidance, LTO = long-term orientation, MAS = masculinity, PRP = personal risk perception, SRP = social risk perception, SER = seriousness, PBI = prevention behavioral intentions.

**Table 9:** The impact of cultural differences on health perception in China.

DV	IV	Beta	t	Sig.
PRP	LTO	0.263	4.023	0.000 ***
SRP	LTO	0.442	7.298	0.000 ***
	PD	−0.194	−3.201	0.002 **
SER	LTO	0.308	4.820	0.000 ***
PBI	LTO	0.450	7.025	0.000 ***
	MAS	−0.134	−2.092	0.038 *

*** *p* < 0.001, ** *p* < 0.01, * *p* < 0.05. PD = power distance, LTO = long-term orientation, MAS = masculinity, PRP = personal risk perception, SRP = social risk perception, SER = seriousness, PBI = prevention behavioral intentions.

## Data Availability

The data presented in this study are available in the reference of this article.
